# Effectiveness of adjuvant occupational therapy in employees with depression: design of a randomized controlled trial

**DOI:** 10.1186/1471-2458-10-558

**Published:** 2010-09-17

**Authors:** Hiske L Hees, Maarten WJ Koeter, Gabe de Vries, Wendy Ooteman, Aart H Schene

**Affiliations:** 1 Department of Psychiatry, Academic Medical Center (AMC), Universiteit van Amsterdam, Meibergdreef 5, 1105 AZ, Amsterdam, The Netherlands; 2 Until November 2008 affiliated with the Department of Psychiatry, Academic Medical Center (AMC), Universiteit van Amsterdam, Meibergdreef 5, 1105 AZ, Amsterdam, The Netherlands

## Abstract

**Background:**

Major depressive disorder is among the medical conditions with the highest negative impact on work outcome. However, little is known regarding evidence-based interventions targeting the improvement of work outcomes in depressed employees. In this paper, the design of a randomized controlled trial is presented in order to evaluate the effectiveness of adjuvant occupational therapy in employees with depression. This occupational intervention is based on an earlier intervention, which was designed and proven effective by our research group, and is the only intervention to date that specifically targets work outcome in depressed employees.

**Methods/Design:**

In a two-arm randomized controlled trial, a total of 117 participants are randomized to either 'care as usual' or *' *care as usual' with the addition of occupational therapy. Patients included in the study are employees who are absent from work due to depression for at least 25% of their contract hours, and who have a possibility of returning to their own or a new job. The occupational intervention consists of six individual sessions, eight group sessions and a work-place visit over a 16-week period. By increasing exposure to the working environment, and by stimulating communication between employer and employee, the occupational intervention aims to enhance self-efficacy and the acquisition of more adaptive coping strategies. Assessments take place at baseline, and at 6, 12, and 18-month follow-ups. Primary outcome measure is work participation (hours of absenteeism and time until work resumption). Secondary outcome measures are work functioning, symptomatology, health-related quality of life, and neurocognitive functioning. In addition, cost-effectiveness is evaluated from a societal perspective. Finally, mechanisms of change (intermediate outcomes) and potential patient-treatment matching variables are investigated.

**Discussion:**

This study hopes to provide valuable knowledge regarding an intervention to treat depression, one of the most common and debilitating diseases of our time. If our intervention is proven (cost-) effective, the personal, economic, and health benefits for both patients and employers are far-reaching.

**Trial registration number:**

NTR2057

## Background

Major depressive disorder (MDD) is among the medical conditions with the highest negative impact on work outcome, even higher than debilitating medical conditions such as rheumatoid arthritis and ischemic heart disease [1]. Depressed employees have an increased risk for both short-term [2-4] and long-term [5] sickness absence, and claim substantially more work disability pensions [6] than their non-depressed colleagues. Out of the ten most common chronic health conditions, depression is associated with the highest reduction in productivity in the workplace [1,7].

Not being able to (fully) participate in the labor market due to depression can lead to subsequent economic and social deprivation [8]. This, in turn, can have an added negative impact on the course of depression [9], initiating a downward spiral. Indeed, studies have demonstrated that employees who have experienced a depression-related disability episode are seven times more likely to have another mental health-related disability episode within 12 months [3,4].

The financial burden of depression-related work disability for society is substantial, and will only continue to increase [8]. In 2000, two-thirds of the total costs associated with depression were work-related ($51.5 out of a total of $83.1 billion) [10]. Even when depressed employees are clinically treated for depression, they still incur 3.2 times higher absenteeism-related costs when compared to non-depressed employees [11]. Moreover, these costs do not include costs related to productivity loss (presenteeism) which are higher than the costs of absenteeism and medical treatment combined [1].

Although the debilitating effects of depression on work-ability have been consistently demonstrated, little is known regarding evidence-based interventions targeting the improvement of occupational functioning in depressed employees [12-14]. In fact, a recent review [13] concluded that our earlier study [15] was the only study that evaluated an intervention specifically focused on occupational functioning in employees with depression. All other studies evaluated the effectiveness of antidepressants and psychotherapy, aimed at symptom reduction, with work as one of the outcome parameters. In this review, insufficient evidence was found for the effectiveness of standard clinical treatment on the improvement of occupational outcomes [13]. These findings are consistent with other studies that have demonstrated that symptomatic improvement does not necessarily correspond to an improvement in workplace performance [16,17]. Instead, functional improvement often lags behind symptom reduction [18,19]. If we want to improve occupational outcomes in depressed employees, we need to develop additional interventions that target not only symptomatic, but also functional improvement.

Based on both scientific literature [20-22] and clinical expertise, we developed an occupational intervention that focuses on work-participation (defined as hours of absenteeism and time until work resumption) in depressed employees. Results from our previous randomized trial [15] evaluating this intervention (mentioned above) were promising: The addition of occupational therapy to standard clinical treatment resulted in a significant reduction in sick-leave days during the first 18 months after baseline assessment. Furthermore, the occupational intervention did not increase work stress, was cost-effective, and was highly regarded by patients. Unfortunately, results demonstrated that adjuvant occupational therapy had no additive effect on the reduction in depressive symptomatology.

Recently, we have developed an improved version of our earlier occupational intervention that is shorter (18 instead of 36 sessions) and which focuses mainly on coping and behavioral change at the workplace. There has also been a shift in the theoretical framework underlying this new occupational intervention. The earlier intervention focused on an extensive period of pre-vocational training before work resumption, an approach that is consistent with the traditional 'train-and-place' model [23]. The new intervention, however, is based on the more recent 'place-and-train' model [24]. Consistent with this latter model, the patient is now encouraged to return to the work environment as soon as possible. In this way, the patient's work situation is directly utilized as an integral component of treatment. First, the return to the work environment can serve as an 'exposure in vivo' to prevent avoidance of problematic work situations. Second, the patient can immediately begin implementing skills (acquired during the intervention) to actively cope with work-related stressors. This increases the patient's positive feelings regarding his or her ability to (fully) resume work. Third, by maintaining contact with the work environment (i.e., with supervisors and colleagues), the 'ranks are kept open': Increased contact with the workplace enhances understanding and social support from colleagues, which facilitates the employee's return to work. Finally, by having so called 'work visits', the new intervention aims to stimulate communication between employer and employee regarding the re-integration process. Previous findings from the physical health field have indicated that early communication between employer and employee lead to improved return-to-work rates [25,26].

Although there is vast evidence that the place-and-train approach is more effective in helping patients return to work than the traditional train-and-place approach [27,28], current research has solely focused on the effect of Individual Placement and Support (IPS), a place-and-train intervention that is specifically designed for unemployed patients with severe mental illness (e.g., schizophrenia). Consequently, work goals in these studies are set lower (working for at least 1 day) than those we seek to achieve in our study. To the best of our knowledge, our new occupational intervention is the first to apply the place-and-train model to improve work-outcome in employees with depression.

In addition to the improved design of the new intervention, the design of this study contains several improvements over our earlier study [15]. First, we are including measures of at-work productivity in addition to measures of work participation. Second, we will expand our sample size in order to increase power, taking into account the increased number of baseline and outcome variables and the small to moderate effect sizes of our earlier study. Finally, we are including personality measures, work-place characteristics (e.g., perception of work load and work problems), and neuropsychological functioning in order to identify potential predictors of treatment effect. Considering that clinical symptoms are hypothesized to be associated with an underlying dysregulation of cognitive processes [29], assessment of neuropsychological functioning might provide a framework for objectifying the cognitive impairments (e.g., reduced concentration, problems with memory and planning) that are frequently reported by patients with depression.

The objective of this paper is to present the design of a randomized controlled trial (RCT) to evaluate the effectiveness of adjuvant occupational therapy (OT), as compared to care as usual (CAU: for description, see Methods), on the work-participation of depressed employees. We hypothesize that patients who are receiving OT have a higher average reduction in hours of absenteeism and a faster return-to-work than patients receiving CAU only. Second, the effectiveness of adjuvant OT regarding work productivity, health-related quality of life, depressive symptomatology, and neurocognitive functioning is evaluated. We hypothesize that patients receiving OT have higher work productivity and a higher quality of life than patients receiving CAU only. Furthermore, we hypothesize no short-term ameliorating effect of adjuvant OT on depressive symptoms or neurocognitive functioning. However, over a more prolonged interval of time, we hypothesize that patients receiving CAU + OT will have a larger reduction in depressive symptoms and neurocognitive functioning than patients receiving CAU only. In addition, we aim to evaluate the cost-effectiveness of OT from a societal perspective. In order to identify the intervention's mechanisms of change (intermediate outcomes), we aim to examine the effect of OT on work-related coping, self-efficacy, and the patient's perception of the work environment. Finally, potential predictors of treatment effect are investigated in order to adequately tailor treatment in the future.

## Methods/Design

### Study design

Our study is a two-arm randomized controlled trial (RCT) to evaluate the effectiveness of adjuvant occupational therapy to regular psychiatric outpatient treatment in patients with depression. Patients are randomized to 'care as usual' (control condition) or 'care as usual' with the addition of occupational therapy (experimental condition). The study includes a baseline assessment and follow-up assessments at 6, 12 and 18 months after start of treatment.

The trial is approved by the Medical Ethics Committee of the Academic Medical Center and the University of Amsterdam. Patients voluntarily participate in the study, and are offered a gift certificate of 35 euro for participation at the last follow-up assessment. A signed informed consent form is obtained from each patient. Participants are informed of their right to withdraw their participation from the study at any time, without specification of reasons or negative consequences for their clinical treatment.

### Inclusion criteria

Participants are eligible for the study if they meet all of the following inclusion criteria: 1) diagnosis of Major Depressive Disorder according to DSM-IV criteria; 2) at least 25% absenteeism due to the depressive disorder; 3) duration of absenteeism of at least 8 weeks *or *the duration of the depressive disorder is at least three months; 4) aged between 18 and 65 years; 5) possibility of returning to their own or a new job; 6) relationship between the depressive disorder and the work situation, i.e.; work is one of the determinants of depressive disorder and contributes substantially (> 25%), *or *depressive symptoms reduce productivity or hinder the return to work; 7) the participant agrees to the possibility of participation in the occupational intervention.

### Exclusion criteria

Participants are ineligible when they meet one or more of the following criteria: 1) diagnosis of bipolar disorder, a psychotic disorder or depression with psychotic characteristics; 2) severe alcohol or drug misuse or dependence; 3) severe physical problems that make participation to the study impossible; 4) severe suicidality; 5) inpatient treatment is indicated; 6) current or recent (i.e., during the current depressive episode) therapy with a psychotherapist, psychologist, or occupational therapist whose content resembles the content of our occupational intervention; 7) current participation in a research study that disables participation in our study.

### Patient recruitment and procedure

Occupational physicians present potential study participants for a telephone screening, where the inclusion- and exclusion criteria are globally assessed by an independent psychiatrist. Next, potential eligible participants for the study receive a standard three-hour psychiatric intake at the outpatient department of the Mood Disorders Program of the Academic Medical Center. In addition, a Structured Clinical Interview for DSM-IV disorders [30] is administered, in order to check whether the participant meets the DSM-IV criteria for a diagnosis of Major Depressive Disorder and fails to meet the DSM-IV criteria of diagnoses mentioned in our exclusion criteria. After the psychiatric intake, eligible patients are asked to sign an informed-consent form. Those who give written informed consent receive the full baseline assessment.

After baseline assessment, participants are randomized to the control or experimental condition. All patients start care as usual; those randomized to the experimental condition also start occupational therapy. During care as usual, the 6-, 12- and 18-month clinical follow-up assessments are conducted by the treating psychiatric resident. When participants have finished care as usual, these assessments are conducted by an independent psychiatrist. Neurocognitive assessments are conducted by a trained researcher. During assessments, participants are asked not to mention their intervention status in order to preserve blinding of the researcher.

### Interventions

#### Occupational intervention

The occupational intervention is provided by two experienced occupational therapists, who have received extensive training in the intervention. The intervention consists of three phases:

#### Phase 1: Problem clarification

The first phase consists of an intake (one session), an occupational anamnesis (three sessions), and a video-observation (one session). During the intake, the patient's current work situation and problem areas are explored. In addition, the patient's treatment goals and expectations regarding the occupational intervention are examined. During the occupational anamnesis, the patient's education and occupational history are systematically analyzed, in order to identify recurrent ineffective coping patterns in stressful situations. During video-observation, the patient is recorded within a simulated work environment (i.e., engaging in role-playing), while the patient performs key tasks relevant to his or her job. Afterward, the recordings are viewed, and the patient's experiences regarding the current tasks, workload, and relationships with colleagues are discussed. In this way, the aspects of the job that the patient experiences as problematic are identified. After completing the problem clarification phase, the occupational therapist discusses the content and goals of the intervention with the occupational physician (OP) by telephone. The therapist also informs the OP that patients are required to work at least 2 hours per week when starting the second phase of the intervention.

#### Phase 2: Occupational intervention

The second phase consists of eight group sessions and four individual sessions. Central to this phase is the 'Quality of Work' (QW) model. The QW model is based on previous literature [20-22] and consists of five factors that affect work performance: 'Work Load', 'Autonomy', 'Relationships at Work' 'Job Perspective', and 'Work-Home Interference'. According to this model, whenever there is an imbalance between work demands and work capacity, problems in occupational functioning are likely to occur. In every group session (approximately eight participants), the QW model is discussed, and patients are taught how to evaluate both the positive and negative factors in their own work situation in accordance with the QW model. In this way, the model provides support for breaking the work problems down into specific, manageable pieces (as opposed to general, insurmountable problems, as perceived by patients). Based on these evaluations, each group member decides what dimension within the model is most important to change in his/her own work situation. This forms the basis for their individual work-reintegration plan. During group sessions, progress regarding each patient's work-reintegration plan is frequently evaluated. In addition, group sessions are used to prepare for the meeting with the employer (through role-playing) and to develop a prevention plan. The advantage of discussing the QW model in a group setting is that patients recognize they are not alone in their problems and that they can benefit from other participants' feedback.

Concurrently with group sessions, three individual sessions and a meeting with the employer take place. During individual sessions, the therapist tries to relate the presently occurring work stressors to the patient's recurrent ineffective coping-pattern (as discussed in the first phase of the intervention). If needed, the therapist provides help with filling out the QW model. In addition, the patient's progress with the work-reintegration plan is monitored during individual sessions.

During the meeting with the patient's employer (i.e., supervisor), the occupational therapist educates the employer regarding the content of the occupational intervention and the consequences of depression for work performance. During this meeting, the patient has the opportunity to openly discuss work-related difficulties with the employer, such as an excessive workload or problematic interpersonal interactions.

#### Phase 3: Follow-up

Within four to six weeks after the completion of the occupational intervention, patients receive a follow-up session to discuss potential problems during the work resumption process.

#### Care as usual

Care as usual consists of treatment by psychiatric residents in the outpatient clinic of the Mood Disorders department at the Academic Medical Center according to a treatment protocol consistent with the APA guidelines [31]. Visits consist of clinical management, including psycho-education, supportive therapy, and cognitive behavioral interventions. Therapies are supervised by an experienced senior psychiatrist on a weekly basis. Pharmacotherapy is started according to a protocolized algorithm. If the patient's condition is deteriorating and outpatient treatment is no longer adequate, the patient may be referred to day treatment or inpatient treatment at the same Mood Disorders department. If the physician wishes to treat in a way that is deviating from the care as usual protocol, he/she is required to contact the research group.

### Randomization

The randomization is conducted by an independent research-assistant, who uses a computerized program based on the minimization-randomization procedure [32]. In order to assess intermediate outcomes and potential predictors of treatment effect, two-thirds of the participants are randomized to the experimental condition, and one-third is randomized to the control condition. Randomization is stratified according to Hamilton score (≤ 17 or ≥18) and number of previous episodes (one or two, vs. three or more).

### Sample size and power

Based on previous results [15], we expect to find a difference of 25% in the main outcome variable between the experimental group and the control group. To achieve a power of 0.80, given a one-sided alpha of 0.05, an estimated effect-size of 0.30, and a control-to-experimental ratio of 1:2, we need at least 35 patients in the control condition and 70 patients in the experimental condition. Considering the high loss to follow-up in this type of research design, we decided to expand the sample size by 10 percent. Thus, the total number of participants included in this study should be at least 116. Power calculations are made with the program G-power [33].

### Primary outcome

#### Work participation

Our primary outcome measure is work participation, defined in terms of absenteeism and work-resumption. The first is operationalized as the average reduction in hours of absenteeism over the three 6-month periods. The latter is operationalized as time until full return-to work (RTW) and time until partial RTW. Time until full (or partial) RTW is defined as the duration of sick leave due to depression in calendar days from the start of treatment until full (or partial) RTW. Full RTW is defined as working the full amount of contract hours in own or other work for at least 4 weeks, without partial or full recurrence. Partial RTW is defined as working an increment of at least 5 hours (when compared to the hours worked at baseline), for at least 4 weeks without partial or full recurrence. Data are derived from diaries that patients keep on a weekly basis during the 18-month study period.

### Secondary outcomes

#### Work productivity

Work productivity is assessed in two ways. First, participants are asked to record weekly their efficiency for the hours worked, on a scale of 1 ('not productive at all') to 10 ('very productive'). Second, the 25-item Work Limitations Questionnaire [34,35] is used to assess productivity loss (presenteeism). The WLQ has demonstrated good reliability (all scales have alpha's above 0.90) and construct validity in both patient and employee populations [35]. In addition to the WLQ total score, we are examining three subscales (the 'Output', 'Time', and 'Mental-Interpersonal' subscale) individually, since previous results have demonstrated a strong association between these subscales and depression-related work limitations [16,36].

#### Depressive symptomatology

Severity of depression is measured with the Hamilton Rating Scale for Depression (HRSD) and the Inventory of Depressive Symptoms (IDS-SR). The 17-item HRSD [37] is a semi-structured clinical interview that covers a range of affective, behavioral and biological symptoms. Scores on this clinician-rated instrument range from 0 to 56, where a score of ≥ 23 is qualified as 'very severe', 19-22 is qualified as 'severe', 14-18 as 'moderate', 8-13 as 'mild', and ≤ 7 as 'normal' [38,39]. The HRSD has acceptable psychometric properties [40]. The IDS-SR [41,42] is a 30-item self-rated instrument to measure the severity of depression, with scores ranging from 0 to 84. A score of ≥ 39 is qualified as 'severe', 30-38 as 'moderate to severe', 22-30 as 'moderate', 14-22 as 'mild' and ≤ 13 as 'normal' [41]. The IDS-SR has good reliability and validity [42,43].

#### Health-related quality of life

Health-related quality of life is measured with the MOS-SF 36 (Medical Outcomes Study-Short Form), a validated and widely used instrument for the assessment of health status [44,45]. This self-report questionnaire contains 36 items regarding behavioral functioning and perceived psychological well-being during the previous four weeks. Responses to the 36 items can be aggregated into eight scale scores. Each scale score can be converted into a 0 to 100 scale, with higher scores indicating higher levels of functioning. The Dutch translation of the MOS-SF 36 has demonstrated adequate psychometric properties [46].

#### Neurocognitive functioning

Speed of information processing is measured with the Trailmaking Test [47,48] part A and the Stroop Color-Word Test [49] part I. Selective attention is measured with the Stroop Color-Word Test Part III, corrected for part II. Divided attention is measured by Trailmaking Test part B, corrected for part A. Working memory is assessed by the subtest 'Letter-Number sequencing' of the WAIS-III [50]. Executive functioning is assessed by the manual version of the Tower of London [51].

### Economic evaluation

Medical consumption is assessed with the Tic-P (Trimbos/iMTA questionnaire for Costs associated with Psychiatric Illness) [52]. The Tic-P is a validated Dutch questionnaire that is commonly used in economic evaluations of treatment in mental-health treatment. In order to calculate the indirect costs of both absenteeism and productivity loss, we are using data from the Work Limitations Questionnaire and weekly self-report diaries (self-constructed).

### Intermediate outcomes: Mechanisms of change

#### Work-related coping

One of the central goals in occupational therapy is to teach patients how to actively cope with the problems they encounter work-related situations. Coping behavior is measured by the Utrecht Coping List (UCL) [53], adapted to specifically address coping behavior in work-related situations. For our study, we are including the UCL-subscales 'Avoidance behavior' (avoiding problematic situations, 8 items), 'Passive reaction' (feeling unable to do anything about the situation, being absorbed by problems, 7 items) and 'Active problem focusing' (approaching problems with confidence and in a goal-directed way, 7 items). All items are rated on a 4-point scale, ranging from 'seldom or never' (coded 0) to 'very frequently' (coded 4).

#### Self-efficacy

In our occupational intervention, an important mechanism of change is to provide patients with more perceived control over their work situation. In other words, our occupational intervention aims to enhance patients' belief in their capacity to deal with work demands, or self-efficacy. Work-related self-efficacy is measured by the 11-item questionnaire 'Expectations regarding work resumption' [54].

#### Perception of the work environment

The 'Perception and Judgment of the Working situation' questionnaire (VBBA) measures the patients' perception of the working situation. The following VBBA subscales are evaluated in order to longitudinally assess changes in perception of the work environment: Work satisfaction, work tempo, work load (mental and emotional), relationships at work (with colleagues and supervisors), and work involvement.

### Potential predictors of treatment effect

To investigate potential predictors of treatment effect, a wide range of variables are included in this study. At baseline, the following variables are measured: Socio-demographic characteristics (e.g., age, education level, gender), clinical history (e.g., severity of depression, number of previous episodes), personality (e.g., neuroticism, extraversion), job characteristics (e.g., type of profession, years of work experience, number of contract hours, contract type) and a retrospective assessment of psychosocial work characteristics four weeks prior to sick leave (e.g., work load, variation in tasks, relationship with supervisor/colleagues). In addition, several aspects within the neurocognitive domain are assessed at baseline as potential predictors of treatment effect: Speed of processing is measured with the Trailmaking Test [47,48] part A and the Stroop Color-Word Test [49] part I. Selective attention is measured with the Stroop Color-Word Test part III, corrected for part II. Divided attention is measured by the Trailmaking Test part B, corrected for part A. Working memory is assessed by the subtest 'Letter-Number sequencing' of the WAIS-III [50]. Executive functioning is assessed by the manual version of the Tower of London [51].

### Statistical analysis

Data are analyzed according to the intention-to-treat principle, i.e., based on original intervention assignment, irrespective of the actual treatments received. Independent samples *t-*tests and χ²-tests are used to evaluate potential differences in baseline characteristics between the intervention and control condition. In case of significant baseline differences between groups, the propensity score method [55,56] is used to adjust for these variables in the evaluations of the treatment effect. Missing independent variables are imputed using multiple imputation techniques.

#### Effect of the intervention

To take into account potential biased outcomes caused by selective loss to follow-up, we use a generalized linear mixed model (GLMM) analysis approach, which, assuming missing at random (MAR) for missing values, gives unbiased effect estimates. MAR is a less restrictive assumption than missing completely at random, and allows loss to follow-up to be related to baseline characteristics that are incorporated in the regression model. GLMM comprises, among others, logistic regression analysis (dichotomous outcomes; e.g., proportion of participants who have returned to work), Poisson regression (count outcomes; e.g., hours worked), Cox regression for censored data (e.g., time to full and partial return to work), and linear regression (continuous outcomes; e.g., scores on depression scales). In all of these analyses, intervention is the independent variable, and a propensity score [55,56] is entered as a covariate to adjust for potential confounders. Since work-productivity and perception of the work environment are conditional upon whether participants have returned to work, a pattern-mixture model analysis is applied for the analysis of these data [57,58].

#### Potential predictors of treatment effect

Predictors are assessed in two ways. First, predictors (e.g., Y) are assessed through the statistical significance of the intervention by Y interaction term. Second, predictors are assessed by using these variables instead of the intervention variable in the mixed model regression analysis restricted to the subgroup that was allocated to the occupational intervention condition.

#### Cost-effectiveness

Cost-effectiveness is assessed from a societal perspective. Therefore, both the direct costs (due to use of mental-health care) and indirect costs (due to production losses and absenteeism) during the 1.5-year follow-up are included. Both direct and indirect costs are estimated according to the Dutch guidelines for cost-analysis in health-care research [59]. The indirect costs are calculated by using the Friction Cost Method [60].

## Discussion

This paper describes the design of a randomized controlled trial to evaluate the effectiveness of adjuvant occupational therapy, as compared to care as usual, on the work-participation of depressed employees. Considering that depression is among the top three disorders with the highest negative impact on work functioning, the lack of interventions targeting the improvement of occupational functioning is noteworthy. Only one previous study [15], also conducted by our research group, has evaluated the effectiveness of an occupational intervention in depressed employees. On the basis of this earlier intervention, we have developed an improved new intervention that focuses on an early return to the work environment and the acquisition of more adaptive coping strategies.

An important strength of our study is the wide range of outcome measures that will be used to evaluate the effectiveness of adjuvant occupational therapy. Few studies have included outcome measures on both the level of work-participation and work-functioning; even fewer studies have included potential predictors of treatment effect. To our knowledge, this is the first study to include measures to explore potential working mechanisms of treatment in this field. This assessment enables us to evaluate a comprehensive picture of the effect of adjuvant occupational therapy in depressed employees. A second strength of the present study is the extensive follow-up period. In their article on improving return-to-work (RTW) research, Pransky and colleagues [61] recommended that "research on RTW requires multiple longitudinal observations in order to fully understand the implications of the effect of an intervention targeting RTW" (p.456).

A possible limitation of our study is the lack of a waiting-list control condition. Thus, in the current study we are unable to compare our findings to a potential improvement in work functioning that might occur during the natural course of depression (i.e., spontaneous recovery). On the other hand, it is considered unethical to deprive patients of evidence-based effective treatment, especially over the course of an 18-month period. Previous studies have demonstrated that chances for relapse increase in untreated depression [62,63]. Another possible limitation of this study is the use of self-report measures for sickness absence data, which may be susceptible to recall bias. In order to minimize bias, patients are asked to record the number of hours worked on a weekly basis. Previous findings have demonstrated that - when reported on a frequent basis - employee records correspond highly with employer records of absenteeism [64].

To conclude, this study hopes to provide valuable knowledge regarding an occupational intervention for treating one of the most common and debilitating diseases of our time. If our intervention is proven (cost-) effective, the personal and economic benefits for both patients and employers are far-reaching.

## Competing interests

The authors declare that they have no competing interests.

## Authors' contributions

AS, MK and WO were applicants on the funding proposal. HH drafted the manuscript and is responsible for the data collection and coordination of the study. GdV designed the occupational intervention protocol. WO contributed to the study's coordination and data collection. AS and MK have commented on earlier versions of the manuscript. All authors have read and approved the final manuscript.

## Pre-publication history

The pre-publication history for this paper can be accessed here:

http://www.biomedcentral.com/1471-2458/10/558/prepub
